# Peripheral Leukocytosis and Clinical Outcomes After Aneurysmal Subarachnoid Hemorrhage

**DOI:** 10.7759/cureus.26778

**Published:** 2022-07-12

**Authors:** Ramesh Shrestha, Sushil Rayamajhi, Sunita Shrestha, Ajit Thakali, Suresh Bishokarma

**Affiliations:** 1 Department of Neurosurgery, Upendra Devkota Memorial National Institute of Neurological and Allied Sciences, Kathmandu, NPL

**Keywords:** glasgow outcome scale, prognosis, ventriculoperitoneal shunt, leukocytosis, early brain damage, aneurysmal subarachnoid hemorrhage

## Abstract

Background: Aneurysmal subarachnoid hemorrhage (SAH) has a high morbidity rate. Following SAH, a powerful systemic inflammatory response ensues contributing to delayed neurological deterioration and outcome. The aim of this study is to investigate if peripheral leukocytosis following SAH impacts clinical outcomes.

Methods: This is a retrospective, observational, single tertiary center study of patients with SAH who underwent microsurgical clipping between 2017 and 2020. The study's inclusion criteria were aneurysmal SAH on baseline computerized tomography (CT), age above 18 years, and hospital admission within 72 hours of bleeding. Traumatic SAH, arteriovenous malformations, and mycotic aneurysms were all excluded. On admission, leukocyte counts were recorded. Demographic and clinical variables were compared between the two groups (TLC ≤12,000 and >12,000). The impact of peripheral leukocytes on clinical outcomes in terms of the Glasgow Outcome Score (GOS) was analyzed. Mann-Whitney U test for continuous variable and Fisher exact test or chi-square test for categorical variables were used for calculation of P-value. A P-value of 0.05 or less was considered statistically significant.

Results: Among 90 patients who underwent clipping of ruptured aneurysms, 40 (44.4%) were anterior communicating artery (ACOMM) aneurysms, and 21 (23.3%) were middle cerebral artery(MCA), and 16 (17.8%) were posterior communicating artery (PCOMM). Clinically 57 patients (63.3%) had a World Federation of Neurosurgical Societies (WFNS) grade 1, 17 patients (18.9%) had a grade 2, four patients (4.4%) had a grade 3, and two patients (2.2%) had a grade 4. On radiological examination, six patients (6.7%) had fisher grade 1, 23 patients (25.6%) had grade 2, 22 patients (24.4%) had grade 3, and 39 (43.4%) had grade 4 SAH distribution. Clinical results were poor in 30 individuals (33.3 %), but good in 60 patients (66.7 %). On admission leukocytosis (>12,000) was seen among 34 (37.8%). Leukocytosis (>12,000) was associated with poor WFNS grade (>2); however, statistical significance was not seen with clinical outcome in terms of GOS.

Conclusion: Poor clinical grade of patients following aneurysmal SAH is associated with peripheral leukocytosis; however, peripheral leukocytosis is not associated with poor outcomes.

## Introduction

Aneurysmal subarachnoid hemorrhage (SAH) has a high morbidity rate, although more and more people are surviving it, with case fatality reducing by 17% over the last three decades and incidence remaining reasonably consistent at nine per 100,000 patient-years [1]. Early brain damage (EBI) has been found to predict clinical outcomes within 72 hours after aneurysmal rupture [2]. The reaction to extravascular blood, poor cerebral autoregulation, the release of products from injured brain tissue, and ischemia-reperfusion injury all contribute to uncontrolled inflammation during EBI [3,4]. Following SAH, there is a powerful systemic inflammatory response involving cytokines and other inflammatory mediators, cellular changes in the CNS parenchymal and peripheral immune cells that peak at 24-48 hours, contributing to delayed neurological deterioration peaks at 24-48 hours, contributing to the delayed neurological deterioration [5]. Increased peripheral monocyte counts and neutrophil to lymphocyte ratios (NLR) are linked to worse outcomes [6,7]. While peripheral leukocytosis is often related to poor clinical grade after aneurysmal SAH, this is not always the case [8]. The purpose of this research is to look into changes in leukocyte counts after SAH and the link between leukocytosis and outcomes.

## Materials and methods

This is a retrospective, observational, single tertiary center study of patients with SAH who underwent microsurgical clipping between 2017 and 2020 at Upendra Devkota Memorial National Institute of Neurological and Allied Sciences at Bansbari, Kathmandu. Ethical approval was obtained from the institutional review board (IRC Number: 119/2022). Inclusion criteria for the study were the presence of aneurysmal SAH on initial computerized tomography (CT), age above 18 years, and admission to hospital within 72 hours of bleeding. Exclusion criteria for the study included: SAH associated with trauma, arteriovenous malformation, or mycotic aneurysms; the presence of diseases and conditions that affect inflammation, such as pregnancy or malignancy [9].

On admission, the total leukocyte count (TLC), monocytes, leukocytes, neutrophils, and basophils were collected. We hypothesized that increased peripheral inflammatory cells would be linked to poor clinical outcomes. Patients’ demographic data, including gender, age, history of smoking, hypertension, diabetes, Hunt-Hess and Fisher grade, an intraventricular extension of blood, the occurrence of hydrocephalus and vasospasm, aneurysm location, duration of hospital stay, and outcome in Glasgow Outcome Score (GOS) were recorded and analyzed. Demographic profile, comorbidities, and clinical and radiological variables were compared between the two groups (TLC ≤12,000 and >12,000) using one- and two-tailed student t-tests. Mann-Whitney U test for continuous variables and Fisher exact test or chi-square test for categorical variables were used for calculation of P-value. A P-value of 0.05 or less was considered statistically significant. SPSS version 20 (IBM, Armonk, NY) was used as a data computation tool.

## Results

**Figure 1:**
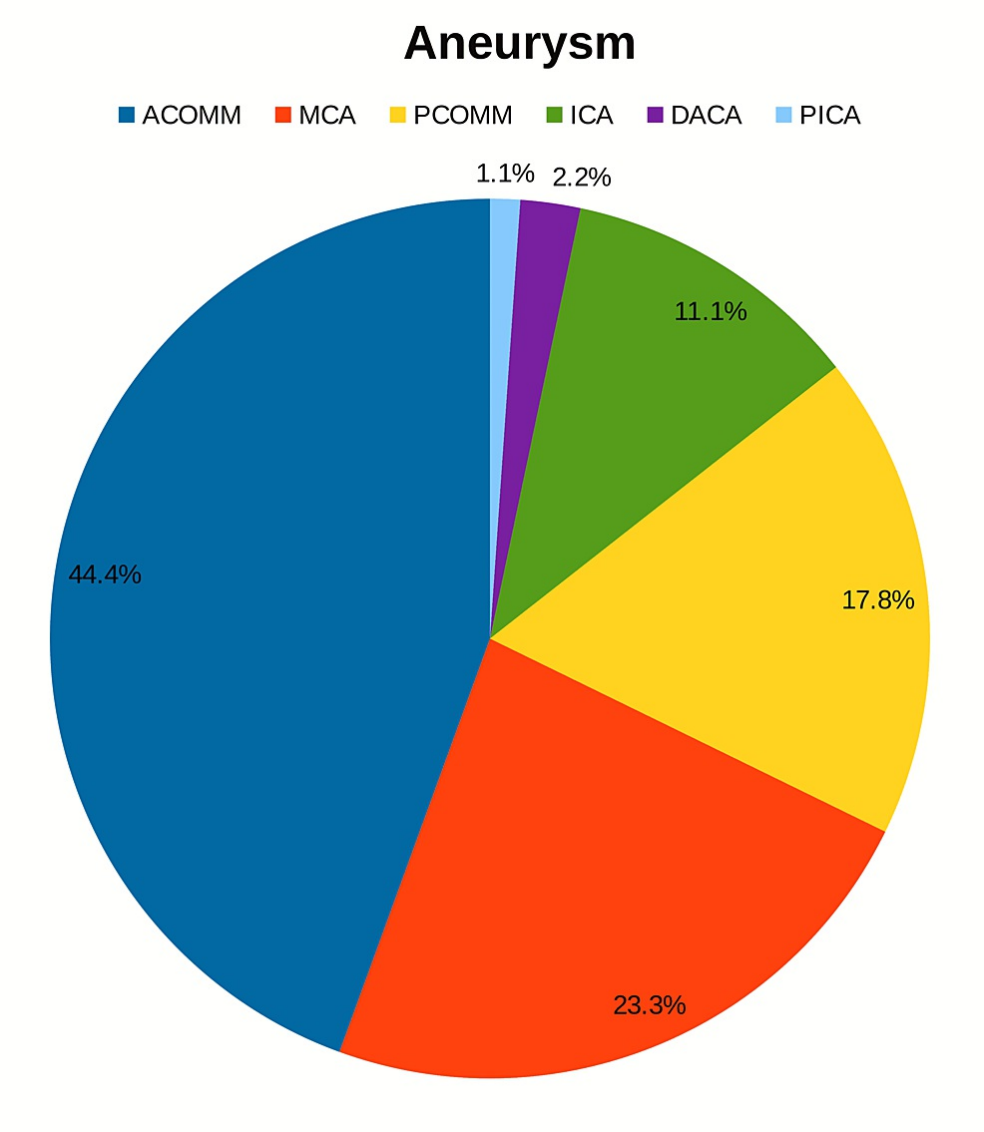
Distribution of aneurysms ACOMM: Anterior Communicating Artery; DACA: Distal Anterior Cerebral Artery Aneurysms; ICA: Internal Carotid Artery; MCA: Middle Cerebral Artery; PCOMM: Posterior Communicating Artery; PICA: Posterior Inferior Cerebellar Artery Aneurysms

The study comprised a total of 90 patients with aneurysmal SAH who underwent microsurgical clipping. Table 1 shows the biological distribution of patient demography. Patients with SAH revealed anterior communicating artery (ACOMM) aneurysms among 40 (44.4%) and middle cerebral artery(MCA) among 21 (23.3%), posterior communicating artery(PCOMM) 16 (17.8%), internal carotid artery (ICA) 10 (11.1%), distal anterior cerebral artery aneurysms (DACA) 2(2.2%) and posterior inferior cerebellar artery aneurysms (PICA) 1(1.1%) (Figure 1). Clinically 63.3% had a World Federation of Neurosurgical Societies (WFNS) grade 1, 18.9% had a grade 2, 4.4% had a grade 3, and 2.2% had a grade 4. On radiological examination, six patients (6.7%) had Fisher grade 1, 23 patients (25.6%) had grade 2, 22 patients (24.4%) had grade 3, and 39 (43.4%) had grade 4 SAH distribution. On admission, leukocytosis (> 12,000) was seen in 34 (37.8%), while 56 (62.2%) had normal leukocyte counts.

**Table 1:** Demographic and clinical profile of aneurysmal subarachnoid patients TLC: Total Leukocyte Count; WFNS: World Federation of Neurosurgical Societies

Characteristics		Total (N=90; 100%)	TLC ≤ 12000 (n=56; 62.2%)	TLC >12,000 (n=34; 37.8%)	P-value
Age (Mean± SD)		53.73±10.86	53.87±10.82	53.5±11.07	0.749
Male		30 (33.3%)	17 (30.4%)	13 (38%)	0.29
Female		60 (66.7%)	39 (69.6%)	21 (61.8%)	
Hypertension		49 (54.4%)	27 (48.2%)	22 (64.7%)	0.96
Diabetes Mellitus		7 (7.8%)	4 (7.1%)	3 (8.8%)	0.53
Smoking		19 (21.1%)	12 (21.4%)	7 (20.6%)	0.57
Headache		81 (90%)	49 (87.5%)	32 (94.1%)	0.26
Vomiting		63 (70%)	37 (66.1%)	26 (76.5%)	0.21
Mean MAP±SD		124±23.56	122.26±23.36	126.21±24.1	0.453
WFNS Grade	1	57 (63.3%)	40 (71.4%)	17 (50%)	0.95
	2	17 (18.9%)	10 (17.9%)	7 (20.6%)	
	3	4 (4.4%)	1 (1.8%)	3 (8.8%)	
	4	2 (2.2%)	5 (8.9%)	5 (14.7%)	
WFNS ≤ 2		74 (82.2%)	50 (89.3%)	24 (70.6%)	0.026
WFNS >2		16 (17.8%)	6 (10.7%)	10 (29.4%)	

Hydrocephalus was found in 23 patients (25.6%), with seven patients (7.8%) requiring rapid external ventricular drain (EVD) placement. Subsequently, six patients (6.7%) required a ventriculoperitoneal shunt (VPS). Likewise, 32 (35.6%) had intraventricular extension of hematoma (Table 2).

**Table 2:** Radiological characteristics of aneurysmal subarachnoid patients ACOMM: Anterior Communicating Artery; DACA: Distal Anterior Cerebral Artery Aneurysms; ICA: Internal Carotid Artery; MCA: Middle Cerebral Artery; PCOMM: Posterior Communicating Artery; PICA: Posterior Inferior Cerebellar Artery Aneurysms; IVE: Intraventricular Extension; EVD: Extradural Ventricular Drainage; TLC: Total Leucocyte Count

Characteristics		Total (N=90; 100%)	TLC≤12,000 (n=56; 62.2%)	TLC>12,000 (n=34; 37.8%)	P-value
ACOMM		40 (44.4%)	23 (41.1%)	17 (50.0%)	0.234
MCA		21 (23.3%)	11 (19.6%)	10 (29.4%)	
PCOMM		16 (17.8%)	13 (23.2%)	3 (8.8%)	
ICA		10 (11.1%)	7 (12.5%)	3 (8.8%)	
DACA		2 (2.2%)	2 (3.6%)	0 (0.0%)	
PICA		1 (1.1%)	0 (0.0%)	1 (2.9%)	
Fisher Grade	1	6 (6.7%)	3 (5.4%)	3 (8.8%)	0.23
	2	23 (25.6%)	16 (28.6%)	7 (20.6%)	
	3	22 (24.4%)	17 (30.4%)	5 (14.7%)	
	4	39 (43.3%)	20 (35.7%)	19 (55.9%)	
Fisher Grade ≤2		29 (32.2%)	19 (34%)	10 (29.4%)	0.419
Fisher Grade >2		61 (67.8%)	37 (66%)	24 (70.6%)	
IVE		32 (35.6%)	17 (30.4%)	15 (44.1%)	0.26
Hydrocephalus		23 (25.6%)	14 (25%)	9 (26.5%)	0.53
EVD		7 (7.8%)	4 (7.1%)	3 (8.8%%)	0.53
VPS		6 (6.7%)	5 (8.9%)	1 (2.9%)	0.26

Vasospasm was developed among 40 patients (44.4%). The mean length of Ictus to hospital stay was 22.37±13.9 days. Thirty patients (33.3%) had poor clinical outcomes, while 60 patients (66.7%) had good clinical outcomes (Table 3). On bivariate analysis, statistical significance was not seen among variables except for dichotomized WFNS (Tables 1-3).

**Table 3:** Outcome of patients following aneurysmal subarachnoid hemorrhage GOS: Glasgow Outcome Scale; TLC: Total Leukocyte Count

Characteristics	Total (N=90; 100%)	TLC ≤ 12,000 (n=56; 62.2%)	TLC >12,000 (n=34; 27.8%)	P- value
Vasospasm	40 (44.4%)	24 (42.9%)	16 (47%)	0.43
Length of Ictus to hospital stay (Mean±SD days)	22.37±13.9	23.78±16.2	20±9.6	0.46
Poor GOS	30 (33.3%)	18 (32.1%)	12 (35.3%)	0.78
Good GOS	60 (66.7%)	38 (67.9%)	22 (64.7%)	

## Discussion

Aneurysmal SAH has a high morbidity rate where early brain injury (EBI) has been shown to predict clinical outcomes within 72 hours of aneurysmal rupture [2,3]. The reaction to extravascular blood, poor cerebral autoregulation, the release of products from injured brain tissue, and ischemia-reperfusion injury all contribute to uncontrolled inflammation during EBI [3,4]. Interleukin-1β (IL-1β) and other pro-inflammatory cytokines cause the release of astrocyte-derived extracellular vesicles into the peripheral circulation and enhance leukocyte transmigration to the central nervous system, resulting in an increase in nuclear factor-kB (NF-kB) activity, which boosts cytokine production in the liver [10]. Following SAH, a powerful systemic inflammatory response peaks at 24-48 hours, contributing to the delayed neurological deterioration [5].

Moreover, the pathogenesis of early and delayed brain injury, as well as the development of vasospasm after SAH, may be influenced by the pro-inflammatory environment in the subarachnoid space [11]. In preclinical models, neutrophil and monocyte levels peaked on day 5 after SAH, and the leukocytes detected in the brain were derived from systemic blood circulation. Even so, the fundamental mechanisms underlying the relationships between leukocytes and outcomes, as well as the relationship between inflammatory cells and EBI, remain unknown [12].

Similar to what we observed regarding WFNS grading in aneurysmal subarachnoid patients, Lagares et al. also found no significant differences in results and were unable to predict significant changes in outcome [13]. However, statistical significance was discovered for dichotomized WFNS where > 2 WFNS had leukocytosis, indicating a poor outcome (Table 1). Furthermore, Degen et al. discovered significant interobserver variability (kappa value of 0.6) for the WFNS scale in a study of 50 patients with aneurysmal SAH [14]. Likewise, the Fisher grading scale, according to Lindvall et al., has a limited predictive value for the outcome of aneurysmal SAH patients due to low specificity and/or sensitivity. We were also unable to obtain a statistically significant result for prognosis prediction [15].

Similarly, Hasan et al. found that 22% of patients developed acute hydrocephalus, and 31% of those patients had ventricular drainage [16]. Hydrocephalus incidence in SAH patients has been documented, ranging from 6% to 67%. In most recent studies, this figure is about 20%-30%, which is close to our results [17]. According to Dupont et al., the degree of hydrocephalus following SAH is a strong predictor of poor functional prognosis regardless of CSF fluid drainage. In our research, no such association was revealed [18].

Furthermore, we studied one-third of the patients with intraventricular extension of hematoma, with 60% having leukocytosis. This finding is corroborated by Nguyen et al. and Zanaty et al., who discovered that intraventricular extension is an independent predictor of SAH cognitive outcomes. However, our result is not statistically significant. Hence, the effect of IVH drainage on SAH is an intriguing area for further research [19,20].

In McGirt et al.'s study, leukocytosis tripled the risk of vasospasm, but we did not find that ratio to be significantly different from one. While poor clinical grade after aneurysmal SAH is associated with peripheral leukocytosis, the opposite is not always true. Despite the fact that several studies have shown that leukocytosis is related to a much longer overall inpatient stay and is an independent risk factor for poor outcomes, we did not discover such findings [8,21].

Bae et al. discovered a mean age of 55.9 ± 11.5 years, which is similar to our finding and, unlike Nguyen et al., has no significant role as a predictor of clinical outcome [20,22]. Similarly, Bae et al. discovered that 40% of the patients had hypertension and 7.5% had diabetes, which is comparable to our demographic findings.

We found a 2:1 ratio of good to poor aneurysmal SAH outcomes in our study. In contrast to Söderholm et al., we found no statistically significant link between high leukocyte count and poor prognosis. This finding may be limited to smokers due to a potential confounder [23]. Cigarette smoking, in addition to the risk factors mentioned above, is the most common modifiable risk factor in patients with SAH (21%). Can et al. found that 45%-75% of patients with ruptured brain aneurysms were current or former smokers, though the study did not describe smoking as contributing to a significant correlation with poor outcomes [24].

ACOMM and MCA aneurysms are the most prevalent causes of aneurysmal SAH (2:1), followed by PCOMM and ICA. This finding is comparable to one discovered by Nisson et al. [25]. In contrast to our findings that aneurysm location has no significant impact on poor clinical outcomes or as a predictor of acute hydrocephalus, Göttsche et al. discovered that aneurysm location can be a significant predictor of acute hydrocephalus and in-hospital mortality, demonstrating the impact of this preexisting biological factor on the course of SAH [26].

As stated by Mank and Brown, other factors that can alter acute leukocytosis in ICU patients include leukemoid reaction, reactive causes, infection, acute allergies, tissue ischemia, drugs/medications, acute hemolysis, and sepsis/septic shock [27]. The impact of such conditions on leukocytosis is considered in the clinical management of SAH patients in ICU. We did not assess in detail such conditions, which may have influenced the leukocyte's response. We consider it as a limitation of our study and suggest considering these factors in future prospective studies.

## Conclusions

Aneurysmal SAH has a high morbidity rate where early brain injury has been shown to predict clinical outcomes. The degree of brain injury is manifested as leukocytosis in response to inflammation caused by hemorrhage. Poor clinical grade in patients after SAH is associated with peripheral leukocytosis. However, peripheral leukocytosis is not associated with poor outcomes. The impact of differential leukocyte counts on outcome could have been studied and hence it is suggested for considering these factors in future studies.
